# Test‐set results can predict participants' development in breast‐screen cancer detection: An observational cohort study

**DOI:** 10.1002/hsr2.2161

**Published:** 2024-06-17

**Authors:** Basel A. Qenam, Tong Li, Abdulaziz Alshabibi, Helen Frazer, Ernest Ekpo, Patrick Brennan

**Affiliations:** ^1^ Medical Image Optimisation and Perception Group, Discipline of Medical Imaging Science, Faculty of Medicine and Health The University of Sydney Camperdown New South Wales Australia; ^2^ Department of Radiological Sciences, College of Applied Medical Sciences King Saud University Riyadh Saudi Arabia; ^3^ The Daffodil Centre The University of Sydney, A Joint Venture with Cancer Council Sydney New South Wales Australia; ^4^ Sydney School of Public Health, Faculty of Medicine and Health University of Sydney Sydney New South Wales Australia; ^5^ Screening and Assessment Service, St Vincent's BreastScreen Fitzroy Victoria Australia; ^6^ Orange Radiology, Laboratories and Research Centre Calabar Nigeria

**Keywords:** breast cancer screening, clinical audit, mammographic test set, radiologist performance

## Abstract

**Background and Aim:**

Test‐sets are standardized assessments used to evaluate reader performance in breast screening. Understanding how test‐set results affect real‐world performance can help refine their use as a quality improvement tool. The aim of this study is to explore if mammographic test‐set results could identify breast‐screening readers who improved their cancer detection in association with test‐set training.

**Methods:**

Test‐set results of 41 participants were linked to their annual cancer detection rate change in two periods oriented around their first test‐set participation year. Correlation tests and a multiple linear regression model investigated the relationship between each metric in the test‐set results and the change in detection rates. Additionally, participants were divided based on their improvement status between the two periods, and Mann–Whitney *U* test was used to determine if the subgroups differed in their test‐set metrics.

**Results:**

Test‐set records indicated multiple significant correlations with the change in breast cancer detection rate: a moderate positive correlation with sensitivity (0.688, *p* < 0.001), a moderate negative correlation with specificity (−0.528, *p* < 0.001), and a low to moderate positive correlation with lesion sensitivity (0.469, *p* = 0.002), and the number of years screen‐reading mammograms (0.365, *p* = 0.02). In addition, the overall regression was statistically significant (*F* (2,38) = 18.456 *p* < 0.001), with an *R*² of 0.493 (adjusted *R*² = 0.466) based on sensitivity (*F* = 27.132, *p* < 0.001) and specificity (*F* = 9.78, *p* = 0.003). Subgrouping the cohort based on the change in cancer detection indicated that the improved group is significantly higher in sensitivity (*p* < 0.001) and lesion sensitivity (*p* = 0.02) but lower in specificity (*p* = 0.003).

**Conclusion:**

Sensitivity and specificity are the strongest test‐set performance measures to predict the change in breast cancer detection in real‐world breast screening settings following test‐set participation.

## 1 INTRODUCTION

In 2020, breast cancer represented a quarter of women's malignancies and one‐seventh of related deaths.^1^ Globally, as cancer incidence has risen in recent decades, mortality rates have also followed.^2^ However, in developed countries, breast cancer mortality has declined^3^ in association with improvements in adjuvant therapy^4^ and the introduction of routine screening mammography since 1988.^5^, ^6^ Multiple studies have indicated that screening is linked to reductions in mortality in the targeted age groups^7^ by at least 20% and up to 48%.^8^, ^9^, ^10^ The literature also shows that the greatest impact of a screening program can take at least 10 years to achieve,^8^ suggesting complexity in the process that requires gaining radiologic expertise on a large scale. For instance, screen‐reading, which involves inspecting and categorizing screening mammograms based on their probability of having breast cancer, is a process that greatly depends on readers' skill indetecting cancer within the technical constraints in imaging techniques and the ambiguity that could be caused by breast anatomy.^11^, ^12^ Thus, screening programs usually implement quality guidelines to achieve expected levels of efficiency and efficacy,^13^, ^14^ and those guidelines are enforced and re‐evaluated through clinical audits.^15^ This enables readers to learn from previous mistakes.^16^, ^17^

Clinical audits are the primary source of feedback for breast screening services rather than radiologists, which makes it difficult for radiologists to identify and mitigate their errors. Thus, researchers have identified the need for mammographic test sets as an alternative assessment method with traits that auditing cannot provide, such as expedited and individualized feedback, and precise evaluation in a controlled environment that is based on preconfirmed diagnoses.^18^ In 1991, the concept was introduced in the United Kingdom by the PERsonal PerFORmance in Mammographic Screening (PERFORMS) self‐evaluation scheme,^19^ which has outlined the test‐set methodology as an educational resource that can provide readers or policymakers with prompt measurement of screen‐reading performance.^18^ In Australia, test‐set‐based research was established by the Breast Screen Reader Assessment Strategy (BREAST) programme, which introduced digital mammography and automated feedback to the test‐set scheme,^20^ thereby enhancing its accessibility and speed. The DetectEDX programme was also introduced in Australia as a cloud‐based test‐set solution that is more portable.^21^ A test set in any of those systems, including BREAST, typically consists of 60 double‐view cases, of which approximately 20 have confirmed malignancies. Participants are asked to inspect the images and point at the lesions in a setup that simulates real‐life breast screening settings. As soon as they complete the test, a modern test‐set platform can provide the participants with case‐by‐case results and summary statistics.

Since obtaining feedback is a major part of the test‐set experience, it is no surprise that an educational value is anticipated. Researchers have examined whether such value could be measured in the form of performance gain in actual screen‐reading. Initial studies indicating evidence of improvement were based on the test‐set history of participants who completed multiple test sets on the same platform, and thus their over‐time results could be compared.^22^, ^23^ To further investigate the improvements, our team inspected the clinical audit results of test‐set participants, and showed that readers improved significantly in audit metrics^24^ including lower rates of recall; higher positive predictive values; higher cancer detection rates. In a subsequent study, focusing on those individuals who read the minimum annual reading numbers (2000 cases) in Australia ^25^ the performances of test‐set participants improved when being compared with their peers who had no experience of test‐set training.^26^ However, it was shown that factors such as reader experience^24^ could affect the extent of change. The current work, focusing on breast cancer detection, aimed to explore performance trends and readers' characteristics that could best describe those who improved most when test‐set training strategies are employed.

## 2 MATERIALS AND METHODS

The study investigated test‐set results of readers who participated in the BREAST test‐set methodology to explore factors that may distinguish readers who have improved their real‐life cancer detection rates in conjunction with test‐set participation from their peers who did not show similar improvements. The test‐set reported performance results include sensitivity, specificity, lesion (location) sensitivity, the receiver operating characteristic (ROC) area under the curve (AUC),^27^ and Jackknife Free‐Response Receiver Operating Characteristic (JAFROC)^28^ Figure of Merit (FOM). Readers' characteristics reported in the test‐set process, including the age when the test set was completed, the number of years screen‐reading mammograms, and the number of years certified as a screen reader were also investigated. Data on gender, fellowship status and number of cases read per week, which also had been collected in the test‐set encounter were excluded from the study because of having high levels of missing values. The cancer detection rate (reported per 10,000 cases) was derived from the annually aggregated clinical audit history of BreastScreen New South Wales (NSW), Australia, which contained data on invasive breast cancer, small invasive breast cancer, and ductal carcinoma in situ (DCIS). The audit data set was linked to readers' corresponding test‐set results using unified identification numbers generated in the data collection process.

### 2.1 Participants

**Figure 1:**
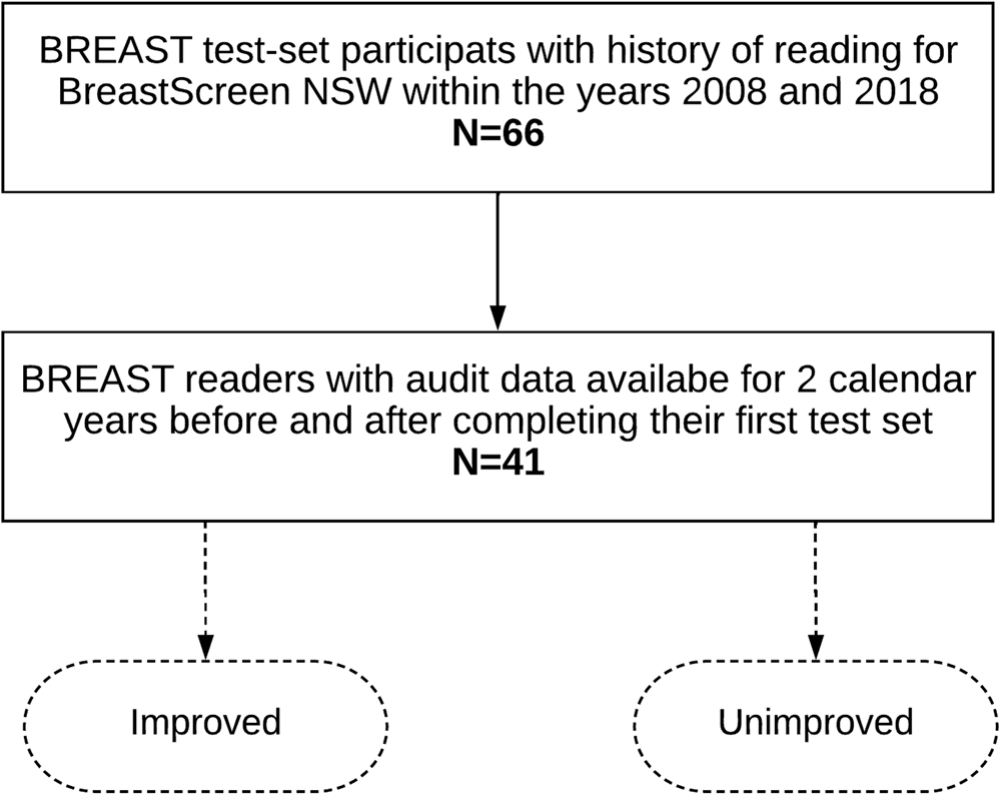
Flowchart of study subject inclusion criteria. Participants were women who had screening mammograms between 2008 and 2018 for BreastScreen NSW. Of the initial group (*N* = 66), only those with audit data available for 2 years before and after their first screening test were included in the final analysis (*N* = 41).

Sixty‐six readers were confirmed to have completed at least one BREAST test set by the end of 2018 (Figure 1) from the total number of 135 BreastScreen NSW readers with clinical audit records between the years 2008 and 2018 inclusive. Of those 66 readers, 41 had audit data for the 2 consecutive calendar years before the year they completed their preliminary test set and for 2 years after it. These numbers excluded participants who were trainees (registrars) at the time of their test‐set encounter. However, as the sample included two breast physicians who had been certified to read for BreastScreen NSW, participants are referred to as readers hereafter, not radiologists. The annual median screen‐reading volume for the cohort across all included years was 3784 with an interquartile range (IQR) of 2799–7822. Since all the study datasets are deidentified, the need for informed consent was waived in the ethics approval process by the Research Ethics Board at the University of XXX under protocol number 2017/142.

### 2.2 Calculation of change in breast cancer detection rate

To define the change in breast cancer detection rate, the clinical audit history of BREAST participants was divided around the calendar year each reader completed their first BREAST test set, and two periods were identified as pretest set and posttest set. Since the availability of readers' annual audit data varied depending on their screen‐reading activity, each period was represented by a 2‐year average. This was to balance between maximizing the number of eligible readers for higher statistical power and including more audit data per reader, especially in the few instances when the detection rate was based on reading fewer than 2000 cases per year. The calendar year between the two periods, in which each reader completed the test set, was excluded from the analysis because it partially represented covered both pre‐ and posttest set performance periods. The detection rates of the two periods were compared using the Wilcoxon signed‐rank test (*α* = 0.05). The detection rate in the posttest set period was then subtracted from the detection rate of the pretest set period to indicate either a positive or a negative value (i.e., the change in detection rate) and based on that each reader was labeled to either the increased performance “improved” or decreased performance” “unimproved” groups as shown in Figure 1.

### 2.3 Analysis of test‐set parameters

The analysis sought to answer the research question from three perspectives. First, Spearman's rank correlation coefficient (*α* = 0.05) was applied to explore potential relationships between the change in cancer detection and the various test‐set parameters. Second, to investigate which parameters could be good predictors of change in cancer detection rate, a multiple linear regression model was built after testing the regression assumptions using the Shapiro‐Wilk test (*α* = 0.05) to inspect the normality of residuals, which indicated that although some of the study variables do not follow the normal distribution, the residuals were normally distributed (*W* = 0.981, *p* = 0.7). Third, each reader was assigned to one of two groups based on the above‐mentioned labels to investigate if readers who increased their detection rate (the improved group) significantly differ in one or more of the test‐set metrics from the readers whose detection rate decreased over time (the unimproved group). Then, the Mann–Whitney *U* test (*α* = 0.05) was used to compare the test‐set metrics. All tests were two‐sided. The statistical analyses were executed via XLSTAT Statistical Software version 2020 4 1, by Addinsoft.

## 3 RESULTS

### 3.1 Overview of readers' breast cancer detection rates

Among the study's 41 participants, there was an increase in the median annual breast cancer detection rate from 55.2 (IQR 46.4–64.4) in the pre‐test set period to 61.6 (IQR 52.9–68.8) per 10,000 cases in the posttest set period (*p* = 0.06). Of those 41 readers, 28 had a positive change in their median detection rate increasing from 52.655 (IQR 47.6–61) in the pre‐test set period to 62.42 (IQR 55.5–73.5) per 10,000 cases in the posttest set period (*p* < 0.001). In comparison, the other 13 readers had a negative change in their median detection rate during the same periods declining from 61.8 (IQR 52.7–77.5) to 54.8 (IQR 42.8–60.9) per 10,000 cases (*p* = 0.01).

### 3.2 Correlation of detection rate and test‐set results

As shown in Table 1, spearman correlation tests, looking into how changes in readers' cancer detection rate related to the reported results metrics of BREAST test sets, indicated moderate positive correlation with sensitivity *r* (40) = 0.688, *p* < 0.001. It also indicated a moderate negative correlation with specificity *r* (40) = −0.528, *p* < 0.001. The data also indicated a low to moderate significant positive correlation with lesion sensitivity *r* (40) = 0.469, *p* = 0.002. Out of all the readers' characteristics assessed, only the number of years screen‐reading mammograms showed a significant correlation with the change in detection rate with *r* (40) = 0.365, *p* = 0.02.

**Table 1:** Spearman correlation tests for the test‐set reported parameters and the change in readers' breast cancer detection rates.

BREAST test‐set variables	Correlation (Spearman)	*p*‐Values (Spearman)
Sensitivity	**0.688**	**< 0.0001**
Specificity	**−0.528**	**< 0.0001**
Lesion sensitivity	**0.469**	**0.002**
JAFROC FOM	0.087	0.58
ROC AUC	0.071	0.66
No. of years screen reading mammograms	**0.365**	**0.025**
Age when taken the test	0.248	0.12
No. of years certified as a screen reader	0.212	0.19

Abbreviations: AUC, area under the curve; BREAST, Breast Screen Reader Assessment Strategy; FOM, Figure of Merit; JAFROC, Jackknife Free‐Response Receiver Operating Characteristic.

### 3.3 Regression analysis

As indicated in the bivariate analysis (Table 1), four parameters, namely ROC AUC, JAFROC FOM, age when the test set was completed, and the number of years certified as a screen reader, did not have significant relationships with the change in detection rate (i.e., no unique variance) and were therefore excluded from the regression analysis. In contrast, lesion sensitivity and the number of years screen‐ reading were significantly related to the detection rate, but they had shared variance with sensitivity and specificity. Thus, a final significant regression equation was found (*F* (2,38) = 18.456 *p* < 0.0001), with an *R*² of 0.493 (adjusted *R*² = 0.466) based on two predictors, sensitivity (*F* = 27.132, *p* < 0.001) and specificity (*F* = 9.78, *p* = 0.003).

### 3.4 Comparison of the subgroups

When readers were divided based on whether their cancer detection rate improved or not before and after test‐set participation, three significant differences were evident in the test‐set results (Table 2). First, sensitivity was higher among the improved group in comparison to the unimproved group (*p* < 0.001). Second, specificity was lower among the improved group in comparison to the unimproved group (*p* = 0.02). Last, lesion sensitivity was higher among the improved group in comparison to the unimproved group (*p* = 0.003).

**Table 2:** Mann–Whitney *U* tests inspecting the differences in test‐set results between the improved and the unimproved readers (in cancer detection rate).

BREAST test‐set variables	Median: Improved group	Median: Unimproved group	*p*‐Value
Sensitivity	92.50	85.00	**0.0001**
Specificity	66.50	83.00	**0.02**
Lesion sensitivity	79.00	61.00	**0.002**
JAFROC FOM	0.755	0.750	0.79
ROC AUC	0.87	0.83	0.62
No. of years screen reading mammograms	18	10	0.08
Age when completed the test	56	51	0.16
No. of years certified as a screen reader	20	16.5	0.18

Abbreviations: AUC, area under the curve; BREAST, Breast Screen Reader Assessment Strategy; FOM, Figure of Merit; JAFROC, Jackknife Free‐Response Receiver Operating Characteristic.

## 4 DISCUSSION

The results of this study show that test‐set reported measures could relate to, predict, and distinguish breast screening readers whose rate of breast cancer detection rate would or would not progress‐ positively in association with test‐set participation. Specifically, three test‐set performance measures (sensitivity, specificity, and location sensitivity) and one experience‐related characteristic (the number of years screen‐reading mammograms) were positively related to change in cancer detection rate. However, specificity decreased as readers improved their detection rates.

The findings described above are not surprising given that specificity is often inversely proportional to sensitivity,^29^ and thus, in any screening test, as one of them increases, it can pull the other down. In mammography, for example, cases are classified based on a probability spectrum that is typically described by a synoptic reporting scale.^30^ The pattern in readers' sensitivity and specificity scores suggests that when they were uncertain, the readers' threshold for classifying a case as cancer or normal was different based on if they improved or did not improve their cancer detection after test‐set participation. This difference in those test‐set performance metrics was also evident in the group comparison (Table 2), where sensitivity, specificity, and lesion sensitivity were major differentiators between the two subgroups. It is important to note that while detecting a cancerous lesion (measured by lesion sensitivity) implies correctly categorizing a cancer case (measured by sensitivity), the opposite is not necessarily true. For instance, it has been shown that readers could recognize the gist of breast cancer in a mammogram^31^ before or without locating the lesion. Nonetheless, in this study's data set, the group that improved in sensitivity did not just classify the images as abnormal based on their gist response, but they were able to accurately localize cancer, showing true improvement in their ability to detect and characterize lesions. While lesion sensitivity was excluded from the regression analysis, it shared variance with sensitivity, meaning that they overlapped in the parts of the variance they could explain. Hence, the final model indicated that sensitivity and specificity alone (i.e., without lesion sensitivity) could explain half of the variance in the change in cancer detection rate after test‐set participation.

Our previous work found that experience was associated with improvements gained after test‐set participation.^24^, ^26^ This is understandable since BREAST test sets were designed to be difficult by including lesions that are challenging to detect, and thus the training is presumably more suited for experienced readers. However, this study's findings were mixed regarding the relationship between experience and increased detection rates. On the one hand, like sensitivity metrics, the number of years screen‐reading mammograms positively correlated with the change in detection rates. On the other hand, the detection rate analyses revealed that readers who improved had a lower baseline detection rate and vice versa. Those results are also reasonable considering that a reader who starts from a lower cancer detection rate would have more room for improvement than a peer whose rate is closer to the high end. The detection rate analyses for the whole study group showed no statistically significant overall change in detection; this may be due to the mix of both the improved and unimproved subgroups, which may have attenuated the power to detect the overall difference in the change. Nonetheless, this finding agrees with the previous studies, which showed that improvements occurred only in the test‐set group and when reading volumes at least met the screening programme requirement of 2000 reads per year.^24^, ^26^ However, since the focus of this study was to investigate the test‐set measures for the improved and the unimproved readers, it was essential not to exclude readers based on a factor that influences performance.^32^, ^33^

The value of test‐set training lies within the premise that it can enhance observer performance, in addition to its validity at measuring it. Therefore, it was critical to establish the relationship between participation in test sets and the development of expertise in image interpretation. This study suggested patterns in readers' test‐set performance that could contribute into their benefiting from the encounter. However, this came with some limitations. First, the study only included eligible test‐set participants from New South Wales, Australia. Second, while the retrospective design allowed for inspecting long‐term trends, it reduced the control over potential confounders. Still, this work provided an important insight that could also guide further research. For instance, future studies could question whether answering a test set with caution (i.e., with lower sensitivity and higher specificity) reduces readers' focus on the learning experience. Furthermore, the regression analysis illustrated that test‐set results could be used to predict and target readers who would benefit the most from test‐set participation. Future research could, in return, explore how test sets could be tailored to benefit readers that current strategies do not favor.

In conclusion, this study showed that sensitivity and specificity are the strongest test‐set performance metrics to indicate how participants' breast cancer detection in real‐world breast screening would change after participation. The difference between the readers who improved and those who did not improve could be rooted in behavioral traits, which could be explored more deeply in future research.

## AUTHOR CONTRIBUTIONS

**Basel A Qenam**: Conceptualization; formal analysis; investigation; methodology; writing—original draft. **Tong Li**: Supervision; writing—review & editing. **Abdulaziz Alshabibi**: Data curation; validation. **Helen Frazer**: Resources; validation. **Ernest Ekpo**: Supervision; writing—review & editing. **Patrick Brennan**: Conceptualization; project administration; supervision; writing—review & editing.

## CONFLICTS OF INTEREST STATEMENT

Patrick Brennan serves as a board member of DetectedX, a medical imaging education company. This institution was not involved in this study in any form. All authors have read and approved the final version of the manuscript. Basel Qenam had full access to all of the data in this study and takes complete responsibility for the integrity of the data and the accuracy of the data analysis.

## TRANSPARENCY STATEMENT

The lead author Basel A. Qenam affirms that this manuscript is an honest, accurate, and transparent account of the study being reported; that no important aspects of the study have been omitted; and that any discrepancies from the study as planned (and, if relevant, registered) have been explained.

## Data Availability

The data that support the findings of this study are available on request from the corresponding author. The data are not publicly available due to privacy or ethical restrictions.
